# Supplementary material for: Effect of myopia on the progression of normal tension glaucoma
Source: PLoS One. 2023 Jun 23;18(6):e0287661. doi: 10.1371/journal.pone.0287661 (PMC10289344; doi:10.1371/journal.pone.0287661)
Supplement: Attachment — Submitted filename: comments 1.docx [file pone.0287661.s002.docx]

**The purpose of the study:**

(Evaluate the risk factors of progression in treated normal-tension glaucoma (NTG) between highly myopic and non-highly myopic eyes.)

- Change (evaluate) to identify or detect risk factors…….change (between) to (in)
- Define in a clear manner (non-highly myopic eyes):
- Is it mild to moderate myopia as it appears from the spherical equivalent in this group of your results?
- The expression (non-high myopia) is not well-defined from another aspect as the term may include emmetropia and hypermetropia also.

**Methods:**

- The sentence (Among 81 eyes from 73 normal tension glaucoma patients ..) is in conflict with the other sentence you mentioned (If both eyes of the same patient were found to be eligible, one eye was randomly selected for analysis.)

Please clarify this point regarding the number of eyes included in the study and if “both eyes of the same patient” were included in the study in some situations.

- Please define the type of tonometers you used in the study.
- You included treated patients in the study, but you did not mention the type of treatment they received either medical, laser, or surgical which may affect the progression of glaucoma. Please explain this point.

**Results:**

- Regarding the baseline data in both groups, the age in the HMG group was significantly less (45.95 ± 9.04 years vs 57.35 ± 10.85 years, p < 0.001)

Did the significant difference in age affect the calculation of risk factors?

- The multivariate Cox proportional hazards model indicated that myopia (HR: 1.2, P=0.019) was risk factor for normal tension glaucoma progression.
- Also in conclusions (Myopia was risk factor for glaucoma progression in treated normal tension glaucoma patients).

Please add (a) before (risk factor)

Which degree of myopia you mean?
