# Supplementary material for: Effect of myopia on the progression of normal tension glaucoma
Source: PLoS One. 2023 Jun 23;18(6):e0287661. doi: 10.1371/journal.pone.0287661 (PMC10289344; doi:10.1371/journal.pone.0287661)
Supplement: Attachment — Submitted filename: Response to reviewers.docx [file pone.0287661.s003.docx]

Letters to reviewers

Dear PLOS ONE Academic Editor and Reviewers:

1. This retrospective cohort study included 41 highly myopic glaucoma (HMG, <-6D) eyes and 40 non-highly myopic glaucoma (NHG,≧-6D) eyes. We change the including eyes to one eye from one patient to avoid bias. We have done the pilot study to calculate the sample size, and the result was 40 patients for each group. The data support the conclusions.
2. We performed univariate and multivariate logistic regression model with SPSS to evaluate the risk factors of high myopic and non-high myopic NTG patients. Subgroup analysis for factors associated with progression of highly myopic and non-highly myopic NTG was also analyzed with logistic regression model. The visual field mean deviation rate of progression in each group was calculated with linear regression model.

5. Reviewer 1#

A. Our study is different from Sohn SW et al‘s study. We divided the participants into 2 groups (non-high myopia and high myopia group with -6 D ), Sohn SW divided into 4 groups. Our study include more high myopia patients (n=42) than Sohn SW (n=24) and we performed subgroup analysis for high myopia group. We also include the total macular layer (TML) thickness asymmetry analysis, which is important for the early diagnosis of high myopia and normal tension glaucoma.

B. Retrospective, observational case series study

C. (1) After the revision, the age difference was not significant. (P=0.07)

(2) After the revision, we include the drug numbers, the percentage of prostaglandin analogue and the percentage of intraocular pressure decrease after treatment

(3) We don’t analysis the effect of systemic treatment for controlling Diabetes mellitus or Hypertension on RNFL thickness evaluated, because the patient numbers with DM or HTN were too small. But we performed subgroup analysis for NTG patients without DM or HTN.

Reviewer 2#

1. Identify risk factors of progression in treated normal-tension glaucoma (NTG) in highly myopic and non-highly myopic eyes.
2. Refractive status was measured via a manifest refraction test using a spherical equivalent.
3. Non-high myopia group include emmetropia and hypermetropia if it’s open angle and normal tension glaucoma
4. After revision, we change to “Among 81 eyes from 81 normal-tension glaucoma patients”, so it’s not conflict with” If both eyes of the same patient were found to be eligible, one eye was randomly selected for analysis.”
5. Non-contact tonometer and i-Care
6. All patients received only medical treatment, and we analyzed the drug numbers, the percentage of prostaglandin analogue and the percentage of intraocular pressure decrease after treatment. When NTG was diagnosed, the patients received Prostaglandin analogue. During the follow-up, if glaucoma progressed or patient can’t tolerate the side effects of Prostaglandin analogue, brimonidine 2mg/ml (Allergan, Inc, Irvine, California, USA) usually was added or replaced.
7. After the revision, the age difference was not significant. (P=0.07)
8. After revision, basal pattern standard deviation (PSD) (OR: 1.55, p=0.016) and post treatment IOP (OR=1.54, p=0.043) were risk factors for visual field progression in normal tension glaucoma patients. The highly-myopic NTG patients evidenced VF progression of 1.37-fold that per 1-D increased myopia. (p=0.388)

6. Yes

Thank you for the comments and I learn a lot.

Kind regards,

Chunmei Hsueh
